# Preparation of Fluconazole **β**-Cyclodextrin Complex Ocuserts:* In Vitro * and * In Vivo* Evaluation

**DOI:** 10.5402/2011/237501

**Published:** 2011-08-25

**Authors:** Hindustan Abdul Ahad, J. Sreeramulu, B. Suma Padmaja, M. Narasimha Reddy, P. Guru Prakash

**Affiliations:** ^1^Department of Pharmaceutics, College of Pharmacy, Sri Krishnadevaraya University, Andhra Pradesh, Anantapur 515003, India; ^2^Analytical Lab, Department of Chemistry, Sri Krishnadevaraya University, Andhra Pradesh, Anantapur 515003, India

## Abstract

The main purpose of the present study was to develop ocuserts of Fluconazole *β*-CD (beta-cyclodextrin) complex and to evaluate both *in vitro* and *in vivo*. Fluconazole was made complex with *β*-CD, and the release rate was controlled by HPMC K_4_M and ethyl cellulose polymers using dibutyl Phthalate as permeability enhancer. Drug-polymer interactions were studied by Fourier transform infrared spectroscopic studies. The formulated ocuserts were tested for physicochemical parameters of *in vitro* release and *in vivo* permeation in rabbits. The optimized formulations (F-5 and F-8) were subjected to stability studies. The formulated ocuserts were found to have good physical characters, thickness, diameter, uniformity in weight, folding endurance, less moisture absorption, and controlled release of drug both *in vitro* and *in vivo*. The optimized formulations retained their characteristics even after stability studies. The study clearly showed that this technique was an effective way of formulating ocuserts for retaining the drug concentration at the intended site of action for a sufficient period of time and to elicit the desired pharmacological response.

## 1. Introduction

Eye drops and eye ointments are conventional ocular dosage forms. They have certain disadvantages like frequent administration, poor availability, massive and unpredictable doses, and drainage of medication by tear/nasolacrimal fluid [1–3]. Ocuserts (ophthalmic inserts) are sterile preparations, with solid or semisolid ingredients with suitable size and shape especially designed for ophthalmic purpose [4–6]. They are mainly composed of a polymeric support with drug (s) incorporated as dispersion or a solution [7–9]. Fluconazole, a synthetic antifungal agent, is a triazole derivative. It is used in the treatment of a wide range of fungal infections [10], and it belongs to class II of biopharmaceutical classification system (BCS) having low water solubility [11]. Cyclodextrins (CDs) are cyclic torus-shaped molecules with a hydrophilic outer surface and a lipophilic central cavity. CDs are available as *α*, *β*, and *γ* forms. Among them *β*-CD is popularly included, which greatly modifies the physical and chemical properties of the drug molecule, mostly in terms of water solubility. Inclusion compounds of Cyclodextrin with hydrophobic molecules are able to penetrate into body tissues; these can be used to release biologically active compounds under specific conditions [12]. It was aimed to prepare ocular films containing Fluconazole *β*-CD complex. 

## 2. Materials and Methods

### 2.1. Materials

Fluconazole was gift samples from Waksman and Selman Pharmaceuticals, Anantapur, India. *β*-CD, acetic acid, and dibutyl phthalate were procured from Merck chemicals, Goa, India. All other reagents and solvents were of analytical grade.

### 2.2. Preparation of Ocuserts

The preparation of ocuserts involved three different steps [13].

#### 2.2.1. Preparation of Drug Reservoir Film

The polymeric drug reservoir films were prepared by dissolving 1.0, 1.5, and 2.0% of HPMC-K_4_M in 15 mL of double distilled water. Along with this 26.95 mg of binary mixture containing Fluconazole *β*-CD was separately dissolved in dilute alkali hydroxide solution and then it was poured to the polymeric solution. The solution was stirred using magnetic stirrer at 100 rpm, and dibutyl phthalate (10% w/w) (which was previously optimized for its concentration) was incorporated (which serves as both plasticizer and permeation enhancer) to the above solution under same stirring conditions. 

After complete mixing the solution was cast in Petri dish (previously lubricated with Glycerin) using a ring of 5.0 cm diameter and with a funnel inverted on the surface (for uniform evaporation of solvent). The cast solution was allowed to evaporate by placing it inside a hot air oven maintained at 37 ± 2°C, 30 ± 0.5% of RH for 24 hours. After drying the medicated films of 8 mm diameter each containing 300 mg of drug were cut using a stainless steel borer, which is previously sterilized. 

#### 2.2.2. Preparation of Rate Controlling Membrane (RCM)

A weighed quantity of ethyl cellulose was dissolved in 10 mL of acetone to obtain 4, 5, and 6% polymeric solutions. Stirring was continuously maintained until the clear solution was obtained. These solutions were poured in Petri dish (previously lubricated with Glycerin) using a ring of 5.0 cm diameter. The solution was evaporated slowly by inverting a glass funnel on a petri dish at room temperature for 12 hours. The dried films were cut into 9 mm diameter using a stainless steel borer.

#### 2.2.3. Sealing

A medicated reservoir disc was sandwiched between two rate controlling membranes. Then this whole unit was placed for 4-5 min over a wire mesh inside the desiccator. Desiccator was previously saturated with ethanol/acetone (60 : 40). This procedure resulted into successful sealing of the medicated reservoir film between two rate controlling membranes. The sealed ocuserts were stored in an airtight container under ambient conditions. 

**Figure 1:**
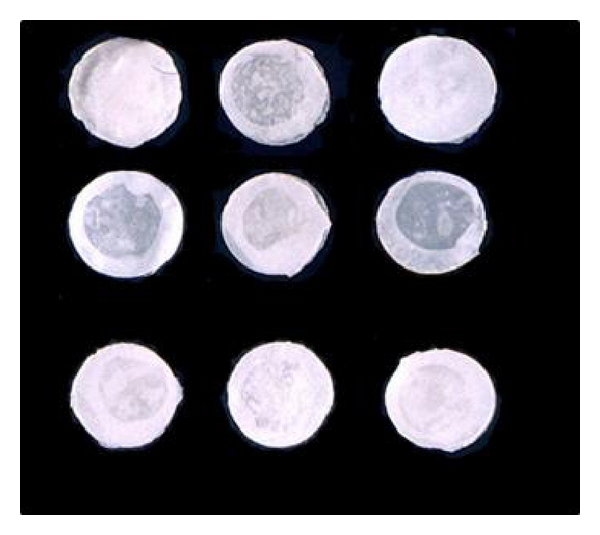
Various formulations of ocuserts.

Plasticizer weight was based on the weight of the polymer. All the above experimentation was carried out under laminar airflow to maintain the sterility conditions of ophthalmic products. The composition of ocusert formulations was represented in Table 1 and shown in Figure 1.

## 3. Evaluation of Polymeric Ocuserts

### 3.1. Compatibility Studies

The compatibility of drug with the excipient used was studied by Fourier transform infrared (FTIR) spectroscopy. The FTIR spectrums of Fluconazole and Formulation (F-5) blend were studied by using FTIR spectrophotometer (Perkin Elmer, spectrum-100, Japan) using the KBr disk method (5.2510 mg sample in 300.2502 mg KBr). The scanning range was 500 to 4000 cm^−1^, and the resolution was 1 cm^−1^. This spectral analysis was employed to check the compatibility of drugs with the polymers used. 

### 3.2. Physical Characterization

The ocuserts were evaluated for their physical characters such as shape, colour, texture, and appearance.

#### 3.2.1. Thickness of Film

Films were evaluated for the thickness using a vernier caliper (For-bro Engineers, Mumbai, India). The average of 5 readings was taken at different points of film, and the mean thickness was calculated. The standard deviations (SDs) in thickness were computed from the mean value [14].

#### 3.2.2. Uniformity in Drug Content

For drug content uniformity, the ocuserts were placed in 5 mL of pH 7.4 phosphate buffer saline and were shaken in orbital shaker incubator at 50 rpm to extract the drug from ocuserts. After incubation for 24 h, the solution was filtered through a 0.45 *μ*m filter and the filtrate was suitably diluted with buffer solution [15, 16]. The absorbance of the resulting solution was measured at 254 nm.

#### 3.2.3. Uniformity of Weight

The weight variation test was carried out using electronic balance (Sartorius GmbH, Gottingen, Germany), by weighing three patches from each formulation. The mean value was calculated, and the standard deviations of weight variation were computed from the mean value.

#### 3.2.4. Folding Endurance

A small strip of ocusert was cut evenly and separately folded at the same place till it breaks. The number of times the ocusert could be folded at the same place without breaking gave the folding endurance [17].

#### 3.2.5. Percentage Moisture Absorption

The percentage moisture absorption test was carried out to check physical stability or integrity of ocular films. Ocular films were weighed and placed in a dessicator containing 100 mL of saturated solution of aluminium chloride, and 79.5% humidity was maintained. After three days the ocular films were taken out and reweighed. The percentage moisture absorption was calculated using the following equation [18]:

(1) $$\begin{array}{cc} & \text{Percentage}\;\text{moisture}\;\text{absorption}\\ & \;\;=\frac{\text{Final}\;\text{weight}-\text{Initial}\;\text{weight}}{\text{Initial}\;\text{weight}}\times 100. \end{array}$$

#### 3.2.6. Percentage Moisture Loss

The percentage moisture loss was carried out to check integrity of the film at dry condition. Ocular films were weighed and kept in a dessicator containing anhydrous calcium chloride. After 3 days, the ocuserts were taken out and reweighed; the percentage moisture loss was calculated using the following equation [19, 20]:

(2) $$\begin{array}{cc} & \text{Percentage}\;\text{moisture}\;\text{loss}\\ & \;\;=\frac{\text{Initial}\;\text{weight}-\text{Final}\;\text{weight}\;}{\text{Initial}\;\text{weight}}\times 100. \end{array}$$

#### 3.2.7. Determination of the Swelling Index and the Surface pH of the Fluconazole Films in Distilled Water

The ocuserts were coated on the lower side with ethyl cellulose (to avoid sticking to the dish) then weighed (*W*_1_) and placed separately in petri dishes containing 25 mL of distilled water. The dishes were stored at room temperature. After 5, 10, 15, 20, 30, 45, and 60 minutes, the films were removed and the excess water on their surface was carefully removed using filter paper. The swollen discs were weighed (*W*_2_), and the percentage of swelling was calculated by the following formula [19–21]:

(3) $$\text{Swelling}\;\text{index}=\frac{W_{2}-W_{1}}{W_{1}}\times 100.$$

The films used for determination of swelling index were used for determination of their surface pH using universal pH paper [22].

### 3.3. In Vitro Drug Release Studies

The ocuserts from each batch were taken and placed in 15 mL vials containing 10 mL of pH 7.4 phosphate buffered saline. The vials were placed in an oscillating water bath at 32 ± 1°C with 25 oscillations per minute. 1 mL of the drug releasing media was withdrawn at various time intervals of 1, 2, 4, 8, 12, 16, and 20 hours and replaced by the same volume of phosphate buffer saline pH 7.4. These samples were filtered through 0.45 *μ*m membrane filter. The filtrate was diluted suitably with the buffer [23, 24]. The drug was estimated in each batch by double beam UV-Vis spectrophotometer (Elico SL 210, Mumbai, India) at 254 nm. The obtained data was treated with mathematical kinetic modeling.

### 3.4. In Vivo Drug Release Study

Out of 5 batches of formulations F-5 and F-8 were taken for *in vivo* study on the basis of *in vitro* drug release studies. The ocuserts were sterilized by using UV radiation before *in vivo* study. The ocusert and other materials were exposed to UV radiation for 1 hour. After sterilization, ocuserts were transferred into polyethylene bag with the help of forceps inside the sterilization chamber itself. The pure Fluconazole that was sterilized along with ocuserts was analyzed for potency by UV spectrophotometer at 254 nm after suitable dilution with pH 7.4 phosphate buffer [25, 26].

Albino rabbits of either sex (New-Zealand strain), weighing between 2.5–3.0 kg, were used for the experiment. The animals were housed on individual cages and customized to laboratory conditions for one day (received free access to food and water). 

The ocuserts containing Fluconazole were taken for *in vivo* study, which were previously sterilized on the day of the experiment and were placed into the lower conjunctival cul-de-sac. The ocuserts were inserted into each of the seven rabbits and at the same time the other eye of seven rabbits served as control.

Ocuserts were removed carefully at 1, 2, 4, 8, 12, 16, and 20 hours and analyzed for drug content as dilution mentioned in drug content uniformity. The drug remaining was subtracted from the initial drug content of ocuserts that will give the amount of drug released in the rabbit eye. Observation for any fall out of the ocuserts was also recorded throughout the experiment. After one week of the washed period the experiment was repeated for two times as before.

### 3.5. Ocular Irritation

The potential ocular irritation and/or damaging effects of the ocusert under test were evaluated by observing them for any redness, inflammation, or increased tear production. Formulation was tested on five rabbits by placing the inserts in the cul-de-sac of the left eye. Both eyes of the rabbits under test were examined for any signs of irritation before treatment and were observed up to 12 hours [27]. 

### 3.6. Stability Studies

Stability testing has become an integral part of formulation development. It generates information on which, proposed for shelf life of drug or dosage forms and their recommended storage conditions are based.

 In the present study, the formulation F-5 was selected for the study, and ocuserts were packed in amber-colored bottles tightly plugged with cotton and capped. They were exposed to various temperatures (60°, 40°, 20°, 10°, and 0°C) for 30 days. At regular intervals, the ocuserts were taken in 5 mL of pH 7.4 phosphate buffer saline and were shaken in orbital shaker incubator at 50 rpm to extract the drug from ocuserts. After incubation for 24 h, the solution was filtered through a 0.45 *μ*m filter, and the filtrate was suitably diluted with buffer solution. The absorbance of the resulting solution was measured at 254 nm [28]. The logarithmic percent of undecomposed drug was plotted against time, and decomposition rate constants (*K*) were obtained at each temperature. The logarithm of decomposition rate constants was plotted against reciprocal of absolute temperature and the resulting line was extrapolated to *K* at 25°C. The shelf life can be obtained by using formula: *T*_90_ = 0.104/*K* at 25°C.

## 4. Results and Discussion

**Figure 2:**
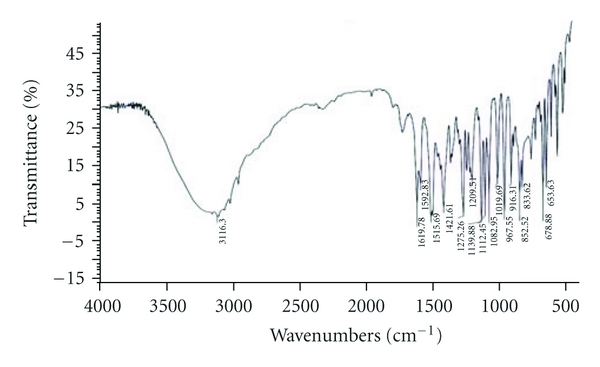
Fluconazole pure drug.

**Figure 3:**
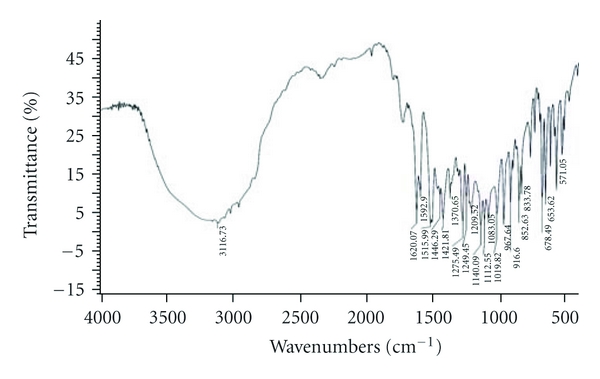
Fluconazole and *β*-CD.

**Figure 4:**
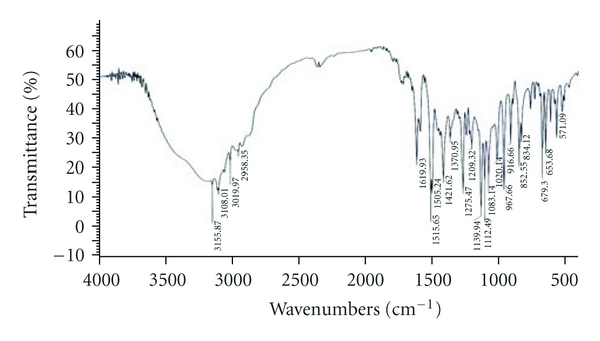
Fluconazole with HPMC.

**Figure 5:**
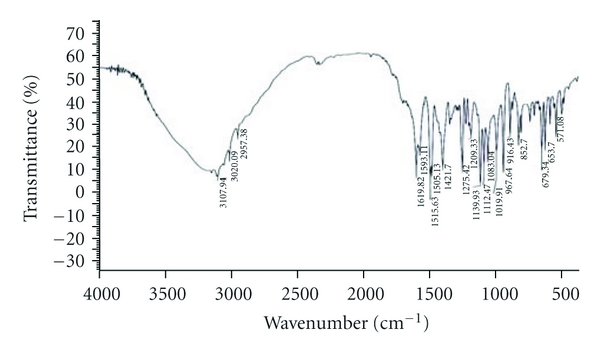
Fluconazole with EC.

**Figure 6:**
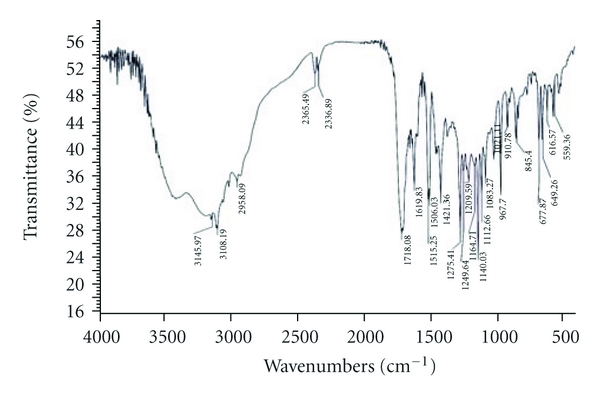
Fluconazole ocusert.

The compatibility of Fluconazole with the polymer used (*β*-Cyclodextrin, HPMC, and EC) was studied by FTIR spectrums (Figures 2, 3, 4, 5, and 6). The characteristic peaks in FTIR spectrums of Fluconazole were seen in the FTIR spectrum of Fluconazole polymer combinations. 

The thickness of the formulated ocuserts was uniform and ranged from 0.16 ± 0.001 to 0.17 ± 0.005 mm. The little variation observed with formulation F-5 might be due to the more concentration of rate controlling membrane. The values of uniformity of weight were found to vary from 15.89 ± 0.028 to 18.48 ± 0.153 mg. All formulations (F-1 to F-9) showed good uniformity in weight. After the moisture loss the ocuserts showed no change in integrity, and it ranged from 6.29 ± 0.109 to 9.68 ± 0.045% and the moisture absorption ranged from 4.78 ± 0.222 to 9.84 ± 0.148%. The highest moisture absorption was marked from formulation F-6 (9.84 ± 0.148%); this may be due to the presence of larger concentration of hydrophilic polymer HPMC-K_4_M. The folding endurance ranged from 74 ± 6.681 to 98 ± 5.621, and no cracks were observed. Formulations F-9, F-3, and F-8 showed maximum folding endurance. The formulated ocuserts were found to have uniformity in drug content: formulation F-4 showed the least drug content (85.65 ± 9.657%) and formulation F-9 showed the highest drug content (97.26 ± 2.255%). The surface pH values of all films were in the range 4.5–6.5. All these values were represented in Table 2.

Water uptake studies were performed for optimized formulations (F-5 and F-8). The water uptake was gradually increasing with time indicating the good wetting nature of the ocuserts. Water uptake values of the formulated ocuserts were shown in Table 3.

**Figure 7:**
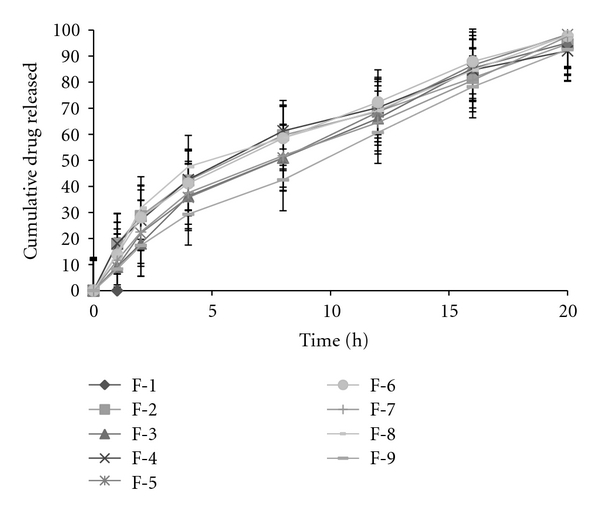
Zero-order plots.

**Figure 8:**
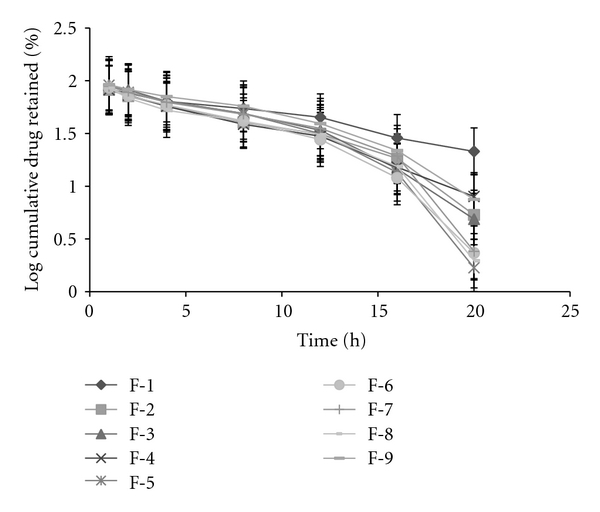
First-order plots.

**Figure 9:**
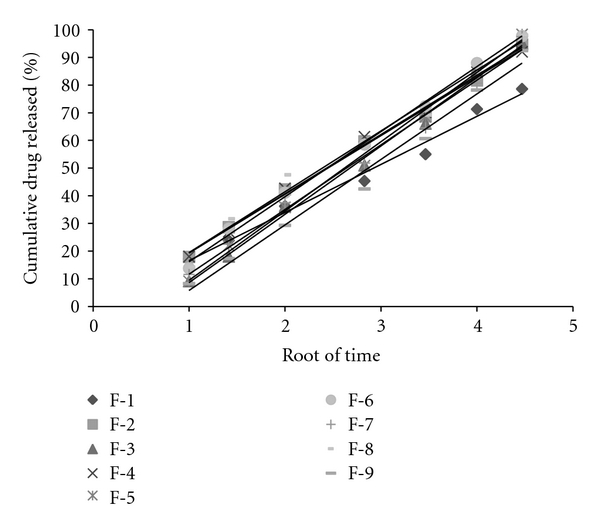
Higuchi's plots.

**Figure 10:**
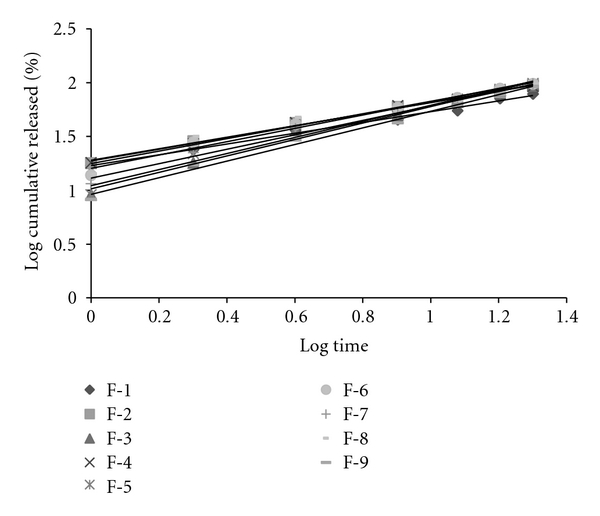
Korsmeyer Peppa's plots.

**Figure 11:**
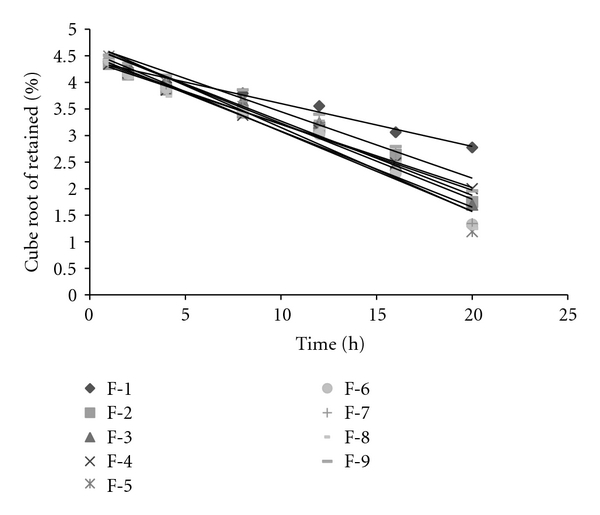
Hixson Crowell's plots.

Based on the highest regression value (*r*), which is near to unity, the formulations F-1, F-2, F-4, F-6, F-8, and F-9 followed Higuchi-Matrix kinetics. This suggests the drug release by swellable polymer matrix through the diffusion of tear fluids. The “*n*” values of formulations F-1 to F-9 were 0.5652, 0.5329, 0.7126, 0.5984, 0.7687, 0.6295, 0.6985, 0.5987, and 0.7748, respectively. This indicates the release by non-Fickian diffusion mechanism. Cumulative percent drug release for F-5 and F-8 was found to be 92.31 and 93.03%, respectively at 20th hour. The kinetic data was tabulated in Tables 4, 5, 6, 7, and 8 and shown in Figures 7, 8, 9, 10, and 11.

**Figure 12:**
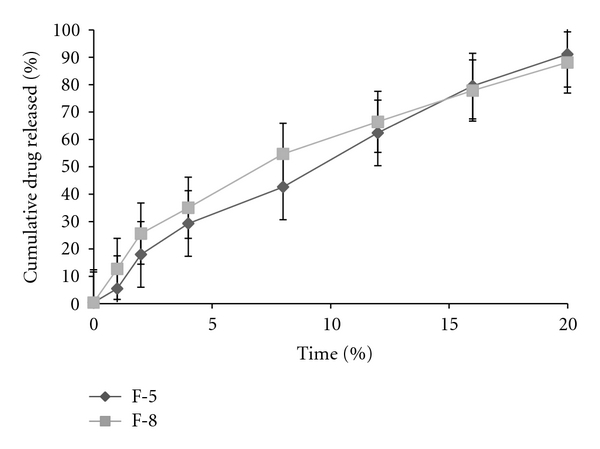
Plots of *in vivo* cumulative drug release versus time for F-5 and F-8.

**Figure 13:**
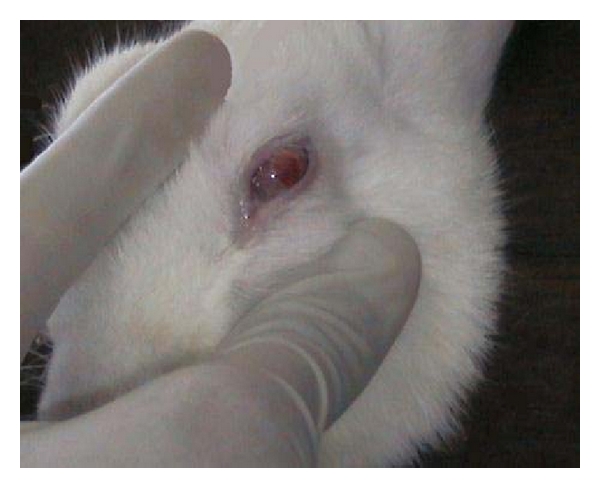
Rabbit with ocusert.

For *in vivo* drug release, formulations F-5 and F-8 were selected based on their uniform drug content and highest *in vitro *drug release. The cumulative percent drug release from F-5 and F-8 was found to be 90.24% and 87.45% at the 20th hour, respectively, which is found to be less when compared to *in vitro* drug release studies. The *in vivo* cumulative drug release versus. time for optimized formulations (F-5 and F-8) was showed in Figure 12. The ocuserts were retained in the rabbit eye during the entire study (Figure 13).

Stability data indicates that the formulations were stable, no major degradation was found (Table 9), and a shelf life of 1.499 years was assigned to the ocuserts (F-8).

## 5. Conclusion

In the present study an attempt was made to develop ocuserts of Fluconazole with improved bioavailability, avoidance of repeated administration and dose reduction. From the experimental finding, it can be concluded that Hydroxy Propyl methyl cellulose is a good film forming hydrophilic polymer and is a promising agent for ocular delivery. Ethyl cellulose was a satisfactory polymeric ingredient to fabricate the rate controlling membrane of the ocusert system. Incorporation of dibutyl phthalate enhances the permeability of Fluconazole, and thus therapeutic levels of the drug could be achieved. Complexation of Fluconazole with *β*-cyclodextrin suggested enhancing the solubility profile of poorly soluble drug Fluconazole and also permeability of the drug through cornea. The kinetic treatment of *in vitro *dissolution data indicated that the ocusert followed non-Fickian diffusion kinetics. *In vivo* release profile indicated that drug release was less compared to *in vitro* release, and there was complete absence of eye irritation and redness of the rabbit eye. The drug remained intact and stable in the ocuserts on storage and shelf life of 1.499 years. Further future work will be progressed to establish the therapeutic utility of these systems by pharmacokinetic and pharmacodynamic studies in human beings.

## Figures and Tables

**Table 1:** Composition of various polymers in different formulations per ring.

Formulation	HPMC-K_4_M (%w/v)	EC (%w/v)	Dibutyl phthalate (%v/w)	Fluconazole *β*-CD (mg)
F-1	1.0	4.0	10.0	300
F-2	1.0	5.0	10.0	300
F-3	1.0	6.0	10.0	300
F-4	1.5	4.0	10.0	300
F-5	1.5	5.0	10.0	300
F-6	1.5	6.0	10.0	300
F-7	2.0	4.0	10.0	300
F-8	2.0	5.0	10.0	300
F-9	2.0	6.0	10.0	300

12 mL of the cast solution was poured into petri dish to prepare circular cast film.

**Table 2:** Physicochemical evaluation of different formulations.

Formulation	Thickness (mm)	Weight uniformity (mg)	Moisture loss (%)	Moisture absorption (%)	Folding endurance	Drug content (%)
F-1	0.16 ± 0.002	17.55 ± 0.107	7.84 ± 0.015	4.78 ± 0.222	74 ± 6.681	89.51 ± 4.568
F-2	0.16 ± 0.001	16.37 ± 0.109	8.54 ± 0.084	5.35 ± 0.155	85 ± 5.847	91.16 ± 6.593
F-3	0.16 ± 0.004	18.35 ± 0.045	9.68 ± 0.045	6.28 ± 0.169	91 ± 6.656	90.26 ± 2.658
F-4	0.16 ± 0.005	15.89 ± 0.028	6.29 ± 0.109	7.84 ± 0.184	68 ± 5.517	85.65 ± 9.657
F-5	0.17 ± 0.003	16.89 ± 0.116	7.57 ± 0.227	8.94 ± 0.167	74 ± 8.594	89.51 ± 7.215
F-6	0.16 ± 0.004	18.48 ± 0.153	8.52 ± 0.024	9.84 ± 0.148	85 ± 6.849	95.21 ± 4.123
F-7	0.16 ± 0.006	15.98 ± 0.117	7.94 ± 0.087	7.51 ± 0.153	85 ± 6.598	88.32 ± 6.597
F-8	0.16 ± 0.005	16.84 ± 0.157	8.54 ± 0.247	8.15 ± 0.048	91 ± 2.955	95.84 ± 5.648
F-9	0.17 ± 0.005	17.97 ± 0.148	9.19 ± 0.028	9.84 ± 0.058	98 ± 5.621	97.26 ± 2.255

All values were expressed as mean ± S.D; number of trials (*n*) = 5.

**Table 3:** Water uptake and swelling behavior.

Time (hours)	Water uptake (mg)	
	F-5	F-8
0	4.51 ± 0.037	4.59 ± 0.253
1	6.29 ± 0.017	5.54 ± 0.214
2	8.45 ± 0.158	7.68 ± 0.314
3	9.79 ± 0.012	10.15 ± 0.168
4	11.54 ± 0.268	12.35 ± 0.247
5	13.57 ± 0.232	16.18 ± 0.658

All values were expressed as mean ± S.D; number of trials (*n*) = 5.

**Table 4:** Kinetic values obtained from zero-order release profile.

Formulation	Slope	Regression coefficient (*r*)	*k* value
F-1	3.5491	0.9056	4.4564
F-2	4.2156	0.9035	5.3535
F-3	4.6656	0.9725	5.2641
F-4	4.2471	0.8946	5.4849
F-5	4.7592	0.9721	5.3749
F-6	4.5875	0.9365	5.5367
F-7	4.5088	0.9358	5.0569
F-8	4.3654	0.9064	5.2698
F-9	4.4845	0.9861	4.8976

**Table 5:** Kinetic values obtained from first order release profile.

Formulation	Slope	Regression coefficient (*r*)	*k* value
F-1	0.0287	0.9851	−0.0721
F-2	0.0265	0.9638	−0.1231
F-3	−0.0659	0.9691	−0.1251
F-4	−0.0535	0.9947	−0.1168
F-5	−0.0059	0.8446	−0.2059
F-6	−0.0735	0.9646	−0.1464
F-7	−0.0651	0.8945	−0.1443
F-8	−0.0655	0.9259	−0.1498
F-9	−0.0559	0.9548	−0.1053

**Table 6:** Kinetic values obtained from Higuchi-matrix release profile.

Formulation	Slope	Regression coefficient (*r*)	*k* value
F-1	18.026	0.9964	15.105
F-2	21.854	0.9934	49.534
F-3	21.489	0.9816	19.779
F-4	20.175	0.9946	19.549
F-5	21.816	0.9847	20.765
F-6	22.168	0.9916	20.146
F-7	22.016	0.9894	20.149
F-8	21.534	0.9916	20.146
F-9	21.146	0.9679	19.243

**Table 7:** Kinetic values obtained from Korsmeyer Peppa's release profile.

Formulation	Slope	Regression coefficient (*r*)	*k* value	*n* value
F-1	0.4998	0.9995	16.489	0.5652
F-2	0.5334	0.99749	18.754	0.5329
F-3	0.7649	0.9967	10.325	0.7126
F-4	0.5502	0.9969	17.984	0.5984
F-5	0.7449	0.9895	10.987	0.7687
F-6	0.6194	0.9946	16.028	0.6295
F-7	0.7743	0.9685	14.987	0.6985
F-8	0.5764	0.9765	17.961	0.5987
F-9	0.7716	0.9962	8.9986	0.7748

**Table 8:** Kinetic values obtained from Hixson Crowell's release profile.

Formulation	Slope	Regression coefficient (*r*)	*k* value
F-1	0.0709	0.9749	−0.0215
F-2	−0.1527	0.9369	−0.0248
F-3	−0.1029	0.9854	−0.0268
F-4	−0.1016	0.9785	−0.0351
F-5	−0.1546	0.9359	−0.0246
F-6	−0.1239	0.9547	−0.0346
F-7	−0.1129	0.9358	−0.0392
F-8	−0.1326	0.9847	−0.0246
F-9	−0.1264	0.9958	−0.0385

**Table 9:** Data obtained from stability studies.

Temp. (°C)	Ab. Temp (*T*)	Rec *T*	D.R.C. (*K*)	Log *K*
60	333	0.00305	0.00164	−2.78516
40	313	0.00319	0.00179	−2.74715
20	293	0.00341	0.00059	−3.22915
10	283	0.00353	0.00036	−3.44369
0	273	0.00366	0.00028	−3.55284
25	298	0.00335	0.00019	−3.72125

Temp: Temperature; Ab. Temp = Absolute Temperature; Rec *T*: reciprocal of absolute temperature; D.R.C. (*K*): decomposition rate constant (Day^−1^); Log *K*: logarithm of decomposition rate constant.
